# Alcohol consumption in Austrian physicians

**DOI:** 10.1186/s12991-019-0246-2

**Published:** 2019-09-24

**Authors:** Edda Pjrek, Leo Silberbauer, Siegfried Kasper, Dietmar Winkler

**Affiliations:** Department of Psychiatry and Psychotherapy, Medical University of Vienna, Währinger Gürtel 18-20, 1090 Vienna, Austria

**Keywords:** Alcohol abuse, Alcohol problem, Alcohol consumption, Problematic drinking, Physicians, Epidemiology

## Abstract

**Background:**

Alcohol is one of the leading exogenous causes for adverse health consequences in Europe. The aim of the present study was to examine the pattern of alcohol consumption in Austrian physicians.

**Methods:**

A telephone survey was conducted in 400 office-based physicians in Austria. Our questionnaire included the four questions of the CAGE questionnaire and questions to assess alcohol consumption on the previous day.

**Results:**

131 participants (32.8%) completed the interview. 3.8% of the subjects had a CAGE score of 2 or higher indicating a problem with alcohol, but this rate was not statistically different from numbers reported for the general population (4.1%). 46.6% of our subjects had drunken alcohol on the previous day. Compared to the general population, the rate of having drunk alcohol yesterday was higher in both gender of our sample, but the amount of alcohol drunk was significantly lower. Doctors in rural areas had drunken alcohol more frequently and in greater quantities on the previous day than those in urban areas. There was a positive correlation between age and the amount of drinking on the previous day, and between age and CAGE scores. Furthermore, subjects who had consumed alcohol yesterday obtained higher scores on the CAGE.

**Conclusions:**

Our findings indicate that the rate of Austrian physicians with problematic alcohol consumption is similar to the general population. Physicians in rural areas and older doctors might be of higher risk for alcohol abuse.

## Background

According to the WHO, alcohol intake in Europe is the highest in the world [1]. Austria is among the top countries in regard to per capita alcohol consumption among the OECD member countries [2, 3] with an estimated 4.9% prevalence of alcohol dependence (7.5% for males and 2.5% for females) in the general population [1]. Alcoholism is a substance abuse disorder that adversely affects virtually every organ system [4]. Patients frequently have poor insight into the disorder and have to be made aware of the negative long-term consequences by their physicians [5].

Problematic drinking of physicians has not only been of scientific interest in the past because doctors with alcohol abuse may provide a lesser standard of care [6] and be more prone to errors [7]. Health professionals play a leading role in alcohol control in the society and their positions are important in the development of health policies [8]. Furthermore, physicians function as role models regarding health behavior [9], and their personal life style and prevention habits are a strong predictor of their counseling and screening of patients for health issues [10]. Finally, medical doctors’ behavior is a marker as how significant certain health problems are perceived in a society [11].

The question whether physicians drink more or less alcohol than the general population has not been answered unanimously. Several studies found elevated rates of problematic drinking [12–16], while others did not report significant differences [17, 18]. However, it is difficult to compare these results due to cultural discrepancies as well as differences in samples, outcome parameters and methodology. In Middle Europe, studies on alcohol drinking in physicians were conducted in Germany [18] and Switzerland [16]. Wurst et al. [19] investigated the prevalence of drinking problems in doctors in one of the nine federal countries of Austria. The present study was aimed to build on these results and to investigate the use of alcohol in physicians in Austria in a nationwide study. Specifically, we intended to investigate whether physicians in Austria showed differences regarding problematic drinking and regarding the amount of alcohol intake compared to published results of the Austrian general population (Austrian Health Survey, *N* = 25,130) [20]. With regard to subgroup analysis, we hypothesized higher rates of problematic drinking and higher alcohol consumption for male physicians, for doctors working in surgical disciplines, and in older physicians.

## Methods

A cross-sectional telephone survey was conducted in a sample of 400 random Austrian office-based physicians. Data of potential subjects were retrieved using the official online directories of the medical chambers of the nine federal states of Austria. The required number of participants from each federal state was calculated according to the number of inhabitants in this state. Participants were selected with equal numbers from medical specialties of the following two subgroups: the conservative disciplines consisted of general medicine, internal medicine, psychiatry, and neurology. The surgical disciplines consisted of surgery, traumatology, orthopedics and gynecology. Half of the doctors worked in private practice, the other half had contracts with health insurances. We ensured an equal sex distribution of study subjects. When one of the participants could not be reached, a replacement was drawn from the same stratum.

Our questionnaire included the CAGE test [21] followed by an assessment of the alcohol consumption on the previous day (yesterday method [22, 23]). These test items were selected to ensure comparability of our results to the Austrian Health Survey [20] that had employed this methodology. The CAGE test is a screening test for problematic drinking and potential alcohol problems, and consists of four yes–no questions (1) Have you ever felt you should Cut down on your drinking? (2) Have people Annoyed you by criticizing your drinking? (3) Have you ever felt bad or Guilty about your drinking? (4) Have you ever had a drink first thing in the morning to steady your nerves or to get rid of a hangover (Eye opener)? CAGE is an acronym for keywords in these questions. A score of two or more yes answers is considered indicative of problematic alcohol use [24]. The CAGE is a valid screening technique with a sensitivity of 93% and a specificity of 76% for the identification of problematic drinking [25]. Alcohol consumption on the previous day was qualitatively and quantitatively inquired, and the results were converted into Austrian standard drinking units (SDU). One Austrian SDU is equivalent to an alcoholic beverage with an ethanol content of 20 g [26]. The assessment of alcohol drinking on the day before the interview has been utilized before to minimize bias of underreporting of alcohol consumption [27].

The offices of the physicians were contacted by telephone on business days at their actual opening hours. We asked for permission to conduct an interview and afterwards explained the purpose of the study. At the request of the study participant, another contact was made at a later date. Statistical analysis was conducted using the R Project for Statistical Computing (version 3.4.3) [28] together with the packages gmodels [29] and reshape2 [30]. Univariate non-parametric tests (Mann–Whitney *U* test, Fisher’s exact test, Chi-squared test, Kendall rank correlation coefficient) were used to examine differences in regard to study subgroups. The *p* ≤ 0.05 level of significance (two-tailed) was adopted. The Bonferroni–Holm correction was applied to the *p* values of comparisons with the Austrian Health Survey, but the subgroup analyses were not corrected because they were explorative in nature. Results are presented as arithmetic mean ± standard deviation.

## Results

Of 400 participants, 131 (32.8%) completed the telephone interview, 73 (18.3%) refused to participate a priori and 196 (49.0%) withdrew their consent to participate after explanation of the objectives of the study or at some later time during the interview (Table 1). Completers did not have a different distribution in terms of gender (*p* = 0.915), geographic location (rural vs. urban) of their medical practice (*p* = 0.585), medical specialty (*χ*^2^ = 1.747, *df* = 7, *p* = 0.972), or presence of contracts with health insurances (*p* = 0.238) compared to the total sample. Mean age was 49.6 ± 6.7 (min.–max.: 38–62) years. The mean CAGE sum score was 0.20 ± 0.59 and five subjects (3.8%) had a score of 2 or higher indicating problematic drinking (CAGE score ≥ 1 in 17 subjects: 13.0%). The rate of subjects with a CAGE score of ≥ 2 was not significantly different from the rate published in the Austrian Health Survey [20] (3.8% vs. 4.1%; *χ*^2^ = 0.027, *df* = 1, *p*_corr_ = 1.000). The rate of elevated CAGE scores was also not different, when analyzing females (1.7% vs. 1.6%; *χ*^2^ = 0.006, *df* = 1, *p*_corr_ = 1.000) and males (5.5% vs. 6.8%; *χ*^2^ = 0.201, *df* = 1, *p*_corr_ = 1.000) separately.

**Table 1:** Demographic properties of the sample of 400 Austrian physicians

Completed interview	131 (32.8%)
No consent for interview	73 (18.3%)
Withdrawal of consent	196 (49.0%)
Completers	
Female:male	58 (44.3%):73 (55.7%)
Rural:urban	22 (16.8%):109 (83.2%)
Conservative:surgical	71 (54.2%):60 (45.8%)
Private:contract with Insurance	68 (51.9%):63 (48.1%)
Age (*μ* + SD, min.–max.)	49.64 ± 6.74 years (38–62)
≤ 49 years:≥ 50 years	69 (52.7%):62 (47.3%)

61 (46.6%) study subjects answered the question about having drunk alcohol yesterday in the affirmative. A comparison of our results with the general population yielded a significantly higher rate of having drunk alcohol yesterday in female (39.7% vs. 15%; *χ*^2^ = 27.652, *df* = 1, *p*_corr_ < 0.0001) and male physicians (52.1% vs. 33%; *χ*^2^ = 11.988, *df* = 1, *p*_corr_ = 0.002). However, the number of SDU was significantly lower in female (0.50 ± 0.68 vs. 1.1; *Z* = − 5.845, *p*_corr_ < 0.0001) and male physicians (0.64 ± 0.71 vs. 1.9; *Z* = − 7.220, *p*_corr_ < 0.0001) compared to the general population.

### Subgroup analyses

The CAGE sum score (0.32 ± 0.95:0.17 ± 0.52; *Z* = − 0.201, *p* = 0.841) and the rate of subjects with CAGE scores ≥ 2 (9.1%:2.8%; *p* = 0.196) were not significantly different by geographical area. However, physicians in rural areas were significantly more likely to have drunk alcohol on the previous day than colleagues in urban areas (rural vs. urban: 68.2%:42.2%; *p* = 0.035) and had also drunk a higher number of SDU (0.86 ± 0.71:0.52 ± 0.69; *Z* = − 2.244, *p* = 0.025).

We did a median split of the sample by age (≤ 49 years vs. ≥ 50 years) and observed that older physicians had higher sum scores on the CAGE (0.35 ± 0.83:0.06 ± 0.24; *Z* = − 2.640, *p* = 0.008) and were more likely to have elevated CAGE scores (8.1%:0.0%; *p* = 0.022). Older doctors had not drunk alcohol more frequently on the day before (44.4%:44.9%; *p* = 0.728) and the number of SDU consumed was not significantly higher than in younger colleagues (0.71 ± 0.84:0.46 ± 0.53; *Z* = − 1.342, *p* = 0.180). However, age was significantly correlated with the number of SDU (*τ* = 0.148. *p* = 0.037) and also with CAGE sum scores (*τ* = 0.228. *p* = 0.002). Subjects, who admitted to having drunk alcohol yesterday, had significantly higher CAGE sum scores (0.38 ± 0.84:0.04 ± 0.20; *Z* = –3.208, *p* = 0.001), and had a higher rate of CAGE scores ≥ 2 (8.2%:0.0%; *p* = 0.020). Furthermore, the number of SDU was higher in subjects with elevated CAGE scores (2.00 ± 0.71:0.52 ± 0.64; *Z* = − 3.578, *p* = 0.0003).

We did not find any statistically significant gender differences regarding the rate of subjects with a CAGE score ≥ 2 (female:male = 1.7%:5.5%; *p* = 0.382), the sum score on the CAGE (0.14 ± 0.48:0.25 ± 0.70; *Z* = − 0.838, *p* = 0.402), the percentage of subjects who had drunk alcohol yesterday (39.7%:52.1%; *p* = 0.164), or the number of SDU consumed on that day (0.50 ± 0.68:0.64 ± 0.71; Z = − 1.259, *p* = 0.208). Regarding subgroup of medical specialty (conservative vs. surgical disciplines) or status of contract with health insurances, we also did not observe any significant subgroup differences on the outcome parameters.

## Discussion

To our best knowledge, this is the first study investigating alcohol consumption in physicians using the yesterday method. Furthermore, this is the first survey on this topic that has employed telephone interviews rather than mailings. A particular strength of this study in contrast to many of the previous reports is the possibility of comparing this sample with a large cohort of the general population. The percentage of Austrian physicians with problematic drinking was not significantly different from the general population. Nevertheless, even a similar percentage can be considered as too high because of physicians’ function as models for health-related behavior and as opinion leaders in public health. Alcohol dependency is a reversible condition with a good prognosis when treated early. The relapse rate for physicians, who have been treated and monitored for substance abuse, is about 2–3 times lower than in the general population [31, 32]. Therefore, it is important to create a climate, where doctors with problematic drinking are identified early and receive treatment rather than having to fear to be subject to disciplinary action.

Medical doctors had about 1.5 (males) to 2.5 (females) times higher rates of alcohol consumption on the previous day than the general population, but consumed about 2 (females) to 3 (males) times lower amounts. We can speculate that due to their demanding jobs doctors are more prone to the attempt of stress regulation with alcohol, but are sufficiently aware of the dangers of the substance to use only smaller quantities. A similar pattern of a higher frequency of drinking but with lower quantities was only reported by McAuliffe et al. [33], but they compared physicians with pharmacists and not with the general population. However, other studies [17, 18] also described lower amounts of alcohol consumed by physicians. When comparing our data with the general population, it is noteworthy that the level of education might influence the consumption of alcohol: Persons with a higher education admitted more frequently to having drank alcohol yesterday (primary school: 30.4%, secondary school 45.5%, university level: 39.3%) and they consumed higher quantities of alcohol in a further analysis of data of the capital Vienna from the Austrian Health Survey [34].

The frequency and quantities of alcohol consumption but not the rate of problematic drinking were higher in physicians with an office in rural than in urban areas. It is noteworthy that no other study in physicians to date has focused on differences regarding this regional differentiation. Increased urbanization was associated with a decline of alcohol consumption on a national level in Austria [35], but the literature on urbanization and alcohol yielded conflicting evidence [36–39]. Moreover, it has become increasingly difficult in many countries to recruit enough physicians, who are prepared to work in the countryside [40, 41]. Working conditions in rural areas might be more stressful for doctors, which could be an important health-related factor influencing alcohol use.

Our results indicate that physicians aged 50 years and above showed problematic drinking more often, which is in line with studies that also recognized older physicians as a risk group [13, 14, 33, 42]. This age trend is unique as alcohol consumption and abuse in the general population show a decrease with age in Austria [20] and elsewhere [43–45]. Our cross-sectional methodology does not give us the opportunity to infer causality, but previous studies hypothesized that younger physicians might have a higher risk-awareness regarding alcohol [46]. However, as these age effects were already described 30 years ago, the persistence of this phenomenon in more recent samples rather suggests that alcohol drinking in older doctors reflects a learned behavior in susceptible individuals undergoing increased work stress [47].

We were unable to ascertain gender differences of alcohol intake in our study. While male doctors had numerically higher alcohol use, this did not reach statistical significance, which might indicate a power problem, but it is also possible that female physicians increasingly adopt male drinking behaviors thus reducing traditional gender differences [48]. A closing female–male gap in alcohol use (gender convergence) has also been reported for the general population during the last 100 years [49]. Interestingly, Austrian doctors from the surgical group were also not more likely to use or abuse alcohol than their colleagues in the conservative group, which is in contrast to the findings of a number of prior studies [14, 15, 18].

It is particularly difficult to get physicians to participate in surveys. Despite adequate methodology, previous studies yielded response rates as low as 6.1% [13] and also the only published study from Austria had a low response rate of 18.6% [19]. Compared to this survey, our study had an about 75% higher response rate (32.8%) probably owing to the interviews by telephone [50]. To achieve higher response rates, future studies investigating physicians should employ multimodal interview techniques, i.e., emails, web-based forms, telephone interviews, include an incentive and seek support of the respective medical association [51, 52]. Despite the fact that participants were not different concerning their demographic properties from the total sample, our survey might still be limited because of the relatively low rate of completed interviews. The particularly high rate of withdrawal of consent itself might be indicative of low openness regarding questions concerning personal drinking habits. In fact, many physicians broke off the interview because they considered the questions about alcohol consumption as “too personal”. As previous studies demonstrated that heavy drinkers are less likely to respond to or refuse to participate in surveys [53, 54], we might suspect that alcohol consumption might be considerably higher in physicians who did not complete the interviews. However, there are also studies that failed to identify differences between responding and non-responding physicians in surveys [55].

To be able to contact physicians of our sample by their business telephone number, we had to conduct the interviews on working days. For a comparison with other studies it is important to keep in mind that Fridays and Saturdays, where alcohol consumption is generally higher [56, 57], were not included as previous days. In the Austrian Health Survey [20], the rates for alcohol consumption on the previous day were about 50.8% on Fridays and Saturdays.

## Conclusions

Our study indicates that physicians in Austria do not show more problematic drinking but consume alcohol more frequently albeit in lower quantities than the general population. Older physicians and those working on the countryside might belong to an at-risk-population. Future studies should be aimed to investigate the reasons underlying this pattern of substance intake.

## Data Availability

The interviewees were promised that no non-aggregated individual data would be published. However, more information can be made available upon reasonable request from the corresponding author as long as anonymity of the interviewees remains guaranteed.

## Abbreviations

CAGE: acronym for Cut down–Annoyed–Guilty–Eye opener
OECD: Organisation for Economic Co-operation and Development
SDU: standard drinking unit
WHO: World Health Organization
